# The effectiveness of introducing detection cameras on compliance with mobile phone and seatbelt laws: a before-after study among drivers in Riyadh, Saudi Arabia

**DOI:** 10.1186/s40621-018-0161-z

**Published:** 2018-08-06

**Authors:** Suliman Alghnam, Jawaher Towhari, Mohamed Alkelya, Abdulaziz Binahmad, Teresa Maria Bell

**Affiliations:** 1 Population Health Section-King Abdullah International Medical Research Center (KAIMRC), King Saud Bin Abdulaziz University for Health Sciences (KSAU-HS), Riyadh, Saudi Arabia; 2 College of Medicine, King Saud Bin Abdulaziz University for Health Sciences (KSAU-HS), Riyadh, Saudi Arabia; 3 Department of Dentistry-King Abdulaziz Medical City, King Saud Bin Abdulaziz University for Health Sciences (KSAU-HS), Riyadh, Saudi Arabia; 4 Center for Outcomes Research in Surgery, School of Medicine, Indiana University, Bloomington, USA

**Keywords:** Road traffic injuries, Motor vehicle crash, Mobile phone, Seatbelt, Detection cameras, Saudi Arabia

## Abstract

**Background:**

Because Saudi Arabia (SA) has struggled with the burden of Road Traffic Injuries (RTIs) for decades, a new automated citations system was implemented in 2018 to improve compliance with seatbelt and mobile phone laws. Therefore, the purpose of this study is to evaluate the impact of the system on the prevalence of seatbelt and mobile phone use among drivers in Riyadh. This is an observational study conducted between 2017 and 2018. A Pre-Post evaluation was employed to determine the impact of a camera detection system on seatbelt and mobile phone use. Two research coordinators collected the observations at several highways and inner intersections around Riyadh (*n* = 3400). We evaluated differences in the prevalence of seatbelt and mobile phone use across the two time periods using a chi-2 test. In addition, we evaluated the association between the new intervention and traffic violation using a logistic regression model.

**Results:**

The overall seatbelt compliance increased significantly from 33.9% (95% CI = 31.7–36.2) to 75.8% (95% CI = 73.7–77.8; *P* < 0.01). In addition, mobile phone use declined significantly from 13.8% (95% CI = 12.2–15.5) to 9.8 (95% CI = 8.8–9.1; *P* < 0.01). We found the detection system to be associated with a significant increase in seatbelt use and also a significant decline in mobile phone use while driving. After implementing the intervention, drivers were 6.1 times (OR = 6.1, 95% CI = 5.2–7.0) more likely to wear seatbelts than before the technology went into effect. Similarly, drivers observed after implementing the cameras were 32% (OR = 0.68, 95% CI = 0.55,0.84) less likely to use mobile phones while driving than those seen prior to the intervention.

**Conclusions:**

This study found a significant reduction in traffic violations following the implementation of a camera detection system in Riyadh. This positive impact is evidence for the role preventative structural strategies play to improve traffic safety and reduce RTI in SA. Therefore, these findings may facilitate further support for policymakers that public health interventions play a significant role to improve traffic safety. Seatbelt and mobile phone use while driving should continue to be monitored, and traffic police may evaluate whether increasing the fine is associated with a significant reduction in traffic violations and associated crashes.

## Background

For decades, Road Traffic Injuries (RTIs) have remained a significant cause of mortality and morbidity in many parts of the world including Saudi Arabia (SA) (Memish et al. 2014). RTIs are the leading cause of Years of Potential Life Lost (YPLL) in SA (Murray and Lopez 2013). Every minute, one traffic crash occurs, and the yearly mortality toll has exceeded 9000 deaths, averaging more than 25 deaths every day (Dawam 2016). Furthermore, it is estimated that 79,000 individuals are disabled due to RTIs and 80% of them suffer permanent disabilities (Saudigazette Newspaper 2018a). Therefore, reducing the burden of RTI has become one of the top priorities of government and health organizations (2015).

Various prevention measures have been implemented globally to decrease the burden of RTIs (Peden 2004). Speeding, seat belt nonuse, and mobile phone use while driving are three common and modifiable risky behaviors associated with a high incidence and severity of RTIs (Jafarpour and Rahimi-Movaghar 2014). Thus, traffic agencies and prevention programs have been targeting their efforts to reduce these factors. This has been manifested by implementing various policies including mandatory seatbelt use and increasing vehicles’ safety (Bendak 2005). Germany decreased its traffic mortality rate by 69% and severity by more than 50% following the implementation of prevention programs (Ernstberger et al. 2015). Similarly, the United States (US) has reported a reduced number of fatalities through lowering speed limit, improving the driving environment, and increasing enforcement of traffic violation (Fred 2016).

Following the path of developed countries, the Saudi traffic police has been trying to implement measures to reduce the incidence and magnitude of RTIs. Despite the existence of written traffic laws and penalties since the early years of motor vehicle use, compliance rates are, unfortunately, very low (Bendak 2007). Seatbelt use for front seat passengers among the Saudi population was reported to be as low as 5% (El Bcheraoui et al. 2015). This estimate is to our knowledge among the lowest reported seatbelt compliance rates worldwide.

In many parts of the world, automated speed detection systems were implemented and have reduced violations and the incidence of RTIs (Kang 2002). Based on previous literature on speed detection cameras, the average reduction in RTIs ranged between 20 and 25% upon implementation (Thomas et al. 2008). As automated speed detection systems proved to be useful in other countries, the Saudi traffic police adopted the initiative in 2010 and introduced a similar system, known as *Saher* (Thomas et al. 2008). Alghnam et al. evaluated health outcomes before and after the implementation of *Saher* and found it associated with a reduction in the likelihood of mortality and severe injuries by 46 and 20%, respectively (Alghnam et al. 2017). These findings suggest that the application of automated detection systems and penalties may improve traffic safety in SA.

Mobile phone use while driving and seatbelt nonuse are two risk factors for RTIs (Klauer et al. 2014). Banning mobile phone use while driving was found associated with reducing injury severity and mortality (Rudisill and Zhu 2017). Due to low compliance among drivers with seatbelt and mobile phone laws, the Saudi traffic police have introduced a new system for automatically issued penalty tickets for mobile phone use while driving and non-seatbelt use (Saudigazette Newspaper 2018b). Special cameras were installed in various locations monitoring compliance and issuing citations. The traffic police department trained and employed 120 individuals to work in the citation headquarter utilizing installed cameras to document and verify violations. After registering and verifying a violation, a text message is sent to the violator including the date and the type of violation. This intervention was initiated gradually at first with an awareness campaign aimed at the public on social media and various television channels. Second, the system was implemented as a trial phase for 2 months with only warning messages to violators (January–February). Finally, the system was officially launched at the beginning of March, 2018. After the new system began, it generated extensive public debate and controversy about its effectiveness (Ministry of Interior 2018c). The system is currently only implemented in three major regions: Riyadh, Jeddah, and Dammam.

Initial and continuous evaluation of policies and systems is essential to guide achieving prevention goals. Our study aims to evaluate the effectiveness of the seatbelt and mobile phone use detection system in terms of reducing the driver’s violations. This evaluation will help provide evidence for policymakers and prevention programs, as well as engage the public to decrease the burden of RTIs in SA. We hypothesize that the intervention is associated with a significant improvement in compliance with traffic laws in SA.

## Methods

This is an observational study among drivers conducted in Riyadh between 2017 and 2018. Riyadh is the capital of Saudi Arabia with an estimated population of 6 million. The highest number of crashes occurs in Riyadh representing 33.7% of all traffic crashes in the country. For this study, we collected data during two-time points: 1) pre-intervention (September–October 2017), and 2) post-intervention (April 2018). Data from the pre-intervention period was collected prior to the announcement of the new system as part of a study to examine the baseline prevalence of seatbelt and mobile phone use among drivers in Riyadh.

Two data collectors visually inspected passing vehicles at several highways and inner intersections around Riyadh. We selected observation sites taking into account clear visibility and the safety of the observers. To capture the variability of traffic flow, the data collectors collected an observation from one lane and then moved to the next. Before selecting a vehicle, the observers specify the vehicle’s features (i.e., color) and then inspect and record the driver’s seatbelt and mobile phone use. Vehicles with tinted frontal glass were excluded and prior to each observation period, observers ran a pilot of five observations to make sure they can clearly see incoming vehicles. Data collectors recorded their observations independently at the same time manually in a serial numbered form. Afterward, the data was entered into Excel 2016 Mac. The observation periods were performed during the evening rush hours (between 15:00 and 18:00 h). At each site, observations lasted about 90-min. For each period (pre and post intervention), data collection lasted about two to three weeks and was conducted on Sundays through Thursdays. Friday and Saturday are the official weekend days in SA. Therefore, data were not collected during the weekend to reduce variability due to different traffic flow.

In this study, we collected observations from four major highways connecting Riyadh to the northern, southern, eastern, and western regions of SA. We collected 200 vehicles from each of the highway sites. While standing on overpasses, data collectors announced that they would observe the third vehicle passing their way.

In addition to collecting observations from major highways, we also observed drivers in various inner intersections throughout the city. Overall, there are nine major zones in Riyadh. Based on size and traffic flow, we selected ten major intersections from each zone. Following that, we selected one intersection randomly from each zone. We used the following abbreviations to refer to the nine sites: EN, ISH, LL, LM, LU, ML, MM, MU, and MUN. The data collectors selected a spot in each of these locations, where it was possible to clearly see passing traffic while ensuring observers’ safety. During the observation periods, the coordinators recorded 100 observations documenting seatbelt and mobile phone use at each intersection.

The primary outcome measure was a mobile phone and seatbelt use among drivers. Mobile phone use was defined as any phone usage while driving including holding it to the ear or holding the device to text, dialing or to taking pictures. After completing data collection, we evaluated agreement between the coordinators using Kappa statistic. There was a substantial agreement between the two observers ranging from 0.70 to 0.96 in mobile phone use and 0.80 to 0.97 in seatbelt use. As such, we selected one observer randomly and used for all analyses in this study.

### Statistical analysis

Statistical analysis was performed using STATA 15 for Mac (STATA Corp., College Station, TX). The prevalence of seatbelt and mobile phone use was calculated with associated 95% confidence intervals (95% CI). In addition, the prevalence of mobile phone use was compared between those who used or did not use a seatbelt. Differences in the prevalence of seatbelt and mobile phone use across the two time periods were evaluated using a Chi-2 test. We used a *p*-value of 0.05, or lower to determine statistical significance.

We evaluated the association between the new intervention and traffic violation using a logistic regression model. The independent variable was whether the observation occurred before or after the introduction of the detection cameras. The dependent variables were seatbelt and mobile phone use. This study was reviewed and approved by the Institutional Review Board (IRB) at King Abdullah International Medical Research center (KAIMRC).

## Results

This study observed 3400 drivers during the two observation periods. The overall seatbelt compliance increased significantly from 33.9% (95% CI = 31.7–36.2) to 75.8% (95% CI = 73.7–77.8, Table 1; *P* < 0.01). In addition, mobile phone use declined significantly from 13.8% (95% CI = 12.2–15.5) to 9.8% (95% CI = 8.5–11.4; *P* < 0.01). On the other hand, mobile phone use was similarly high among individuals who did not wear seatbelts across the two periods (pre-intervention = 17.7%, post-intervention = 16.8%, *P* = 0.6).

**Table 1:** Comparison of the prevalence of seatbelt and mobile phone use among drivers in the two observation periods

Location	Seatbelt Use % [95%CI]		Mobile Phone Use % [95%CI]	
	Pre-cameras	Post-cameras	Pre-cameras	Post-cameras
Overall (*N* = 3400)	**33.94% [31.72–36.22]**	**75.82% [73.72–77.80]**	**13.82% [12.26–15.54]**	**9.88% [8.55–11.39]**
Highways (*N* = 1600)	**45.25% [41.82–48.72]**	**89.25% [86.90–91.21]**	**15.12% [12.80–17.78]**	**9.25% [7.42–11.46]**
East	**36.00% [29.59–42.94]**	**85.50% [79.85–89.76]**	19.00% [14.10–25.09]	13.00% [8.97–18.46]
North	**46.50% [39.63–53.49]**	**91.50 [86.70–94.67]**	**17.00% [12.37–22.90]**	**5.50% [3.05–9.70]**
West	**40.50% [33.86–47.50]**	**86.00% [80.41–90.18]**	**17.50% [12.80–23.45]**	**9.50% [6.11–14.46]**
South	**58.00% [50.98–64.70]**	**94.00% [89.68–96.57]**	7.00% [4.17–11.51]	9.00% [5.72–13.88]
Inner Intersection (*N* = 1800)	**23.88% [21.21–26.79]**	**63.88% [60.68–66.96]**	12.66% [10.64–15.00]	10.44% [8.60–12.62]
EN	**13.00% [7.62–21.28]**	**55.00% [45.01–64.60]**	17.00% [10.75–25.83]	11.00% [6.13–18.94]
ISH	**21.00% [14.00–30.25]**	**39.00% [29.81–49.03]**	19.00% [12.36–28.05]	14.00% [8.39–22.43]
LL	**11.00% [6.13–18.94]**	**50.00% [40.15–59.84]**	15.00% [9.16–23.57]	14.00% [8.39–22.43]
LM	**27.00% [19.09–36.69]**	**81.00% [71.94–87.63]**	6.00% [2.68–12.87]	8.00% [4.00–15.35]
LU	**15.00% [9.16–23.57]**	**49.00% [39.19–58.87]**	9.00% [4.69–16.56]	18.00% [11.55–26.94]
ML	**53.00% [43.05–62.70]**	**90.00% [82.23–94.59]**	8.00% [4.00–15.35]	4.00% [1.48–10.33]
MM	**24.00% [16.52–33.49]**	**84.00% [75.29–90.04]**	10.00% [5.40–17.76]	8.00% [4.00–15.35]
MU	**30.00% [21.71–39.84]**	**84.00% [75.29–90.04]**	13.00% [7.62–21.28]	8.00% [4.00–15.35]
MUN	**21.00% [14.00–30.25]**	**43.00% [33.52–53.02]**	17.00% [10.75–25.83]	9.00% [4.69–16.56]

Bold is significant at the 0.05 level

A higher increase in the prevalence of seatbelt use was observed at highway locations than the overall sample. Seatbelt use increased from 45% (95% CI = 41.8–48.7) among drivers to 89.5% (95% CI = 86.9–91.2) after implementing the detection cameras. Furthermore, in the period after the technology was launched, seatbelt use varied between 85.5% (95% CI = 79.8–89.7) and 94.0% (95% CI = 89.6–96.5). Likewise, the overall prevalence of mobile phone use declined significantly from 15.1% (95% CI = 12.8–17.7) to 9.2% (95% CI = 7.4–11.4) and ranged between 5.5% (95% CI = 3.0–9.7) and 13.0% (95% CI = 8.9–18.4).

At inner intersections, seatbelt use increased significantly after implementing detection cameras but was lower than what was observed at highways (63.8%, 95% CI = 60.6–66.9). The estimated prevalence of seatbelt use ranged between 39.0% (95% CI = 29.8–49.0) and 90% (95% CI = 82.2–94.5). About 10.4% (95% CI = 8.6–12.6) of drivers observed at inner intersections were using mobile phones while driving. The prevalence of mobile phone use ranged between 4.0% (95% CI = 1.4–10.3) and 18.0% (95% CI = 11.5–26.9).

According to the regression analysis, we found the detection system to be associated with a significant increase in seatbelt use and also a substantial decline in mobile phone use while driving. After implementing the intervention, drivers were six times (OR = 6.1 95% CI = 5.2–7.0) more likely to wear seatbelts than before the technology went into effect. Likewise, drivers observed after implementing the cameras were 32% (OR = 0.68, 95% CI = 0.55,0.84) less likely to use mobile phones while driving than those seen before detection cameras (Table 2). Additionally, we stratified the analysis by location and found the impact of detection camera on seatbelt use to be more pronounced at highway locations (OR = 10.0, 95% CI = 7.7–13.0) than at inner intersections (OR = 5.6, 95% CI = 4.6–6.9). While mobile phone use did not differ between the two periods in inner intersections, after implementing the cameras, drivers at highway locations were 43% (OR = 0.57, 95% CI = 0.42,0.77) less likely to use a mobile phone than before implementing the law.

**Table 2:** Logistic regression analyses of the association between the new camera detection system and wearing seatbelts and mobile phone use while driving

Variables	Seatbelt use OR (95% CI)	Mobile phone use while driving OR (95% CI)
Overall (*N* = 3400)		
Pre-camera	Reference	Reference
Post-camera	**6.10 (5.25–7.08)**	**0.68 (0.55–0.84)**
Highways (*N* = 1600)		
Pre-camera	Reference	Reference
Post-camera	**10.04 (7.71–13.07)**	**0.57 (0.42–0.77)**
Inner intersections (*N* = 1800)		
Pre-camera	Reference	Reference
Post-camera	**5.63 (4.60–6.91)**	0.80 (0.60–1.07)

Bold is significant at the 0.05 level

## Discussion

This study found a significant reduction in traffic violations following the implementation of a camera detection system in Riyadh. If the present results are sustained, we are likely to see reductions in preventable RTIs over time. Previous studies from the US have found laws aimed to reduce distracted driving to be associated with around 8% in fatal crashes (Lim and Chi 2013). Furthermore, a global study of traffic law enforcement suggests that recent seatbelt and helmet safety programs in middle-income countries were associated with reduced fatalities (Urie et al. 2016). The improved compliance we present here is a testament to the role preventative strategies play to reduce traffic violations and their associated RTIs. However, seatbelt and mobile phone use levels remain worse than developed countries despite the positive results reported here. These finding may facilitate further support from policymakers and engage the public in advocacy for traffic safety. Clearly, there is a need for further investments in safety interventions to reduce the burden of traffic crashes on population health.

Our findings are in line with earlier literature from developed countries suggesting a positive effect of automated systems in improving traffic safety (Li et al. 2013; Mendivil et al. 2012; Skubic et al. 2014). Specifically, studies from the US that investigated the impact of red light and speed detection systems have found significant positive effects following their implementation (Retting and Kyrychenko 2002; Shin et al. 2009).

A number of factors may explain the positive impact of detection cameras on traffic violations presented in this study. First, drivers may have changed their behavior to avoid citations. A previous study evaluated the effect of introducing red light detection camera on drivers’ behavior and found significant change represented by reduced violations (Wahl et al. 2010). Other factors that may have amplified the drivers’ response to the new intervention are financial factors. Despite being a high-income country, the new financial policies in 2018, represented by the introduction of sales tax for the first time and increased energy prices, may have played a role in drivers being more sensitive to financial citations and, thus, change their behavior (Alghannam 2018). Finally, because the study collected observations from two-time points, another potential explanation is regression to the mean phenomenon (Brenac 2010).

The reduction of mobile phone use after the implementation of detection cameras remains suboptimal compared to the prevalence in developed countries. Previous literature suggests that 2.2% of UK drivers, 2.8% Canadian drivers, and 7% of US drivers report using a mobile phone behind the wheel (Burns et al. 2008; Sullman 2012; Wenners et al. 2013). A study in New York state by McCart et al. examined the impact of new legislation mandating hands-free devices and found a significant reduction in mobile phone use from 2.3 to 1.1% (McCartt et al. 2003). In SA, there is a need for further research and investment in public health interventions to reduce mobile phone use to levels that are similar to developed countries.

The present data suggested that implementing detection cameras did not reduce mobile phone use in inner-city intersections. This is despite the fact that the traffic police did not specify the locations for implemented cameras. Drivers may have been less likely to change their behavior because they perceived that enforcement was limited to major roads. In addition, smartphones, with driving features such as navigation, may lure drivers to violate the law despite the existence of detection systems (Staver 2018; Young 2018). It could also be the case that, unlike seatbelts, where you wear it once in the vehicle, drivers feel they are less likely to get caught using a mobile phone than failing to comply with the seatbelt law. This, in turn, makes drivers underestimate the risk of receiving a citation and, as a result, less likely to change their behavior. Previous studies suggest that high visibility police enforcement is associated with a significant reduction in mobile phone use while driving (Cosgrove et al. 2010) Police enforcement may consider this for future interventions to reduce the prevalence of traffic violations.

Seatbelt compliance increased significantly according to our findings, which consistent with what has been reported in other countries. As part of the Bloomberg Global Safety Program, public health interventions in Russia were associated with increasing seatbelt compliance from 47.5 to 88.8% (Slyunkina et al. 2013). In South Korea, a national intervention including awareness campaign and doubling seatbelt fines increased compliance from 23% in 2000 to around 98% in late 2001 (Yang and Kim 2003). Despite this positive finding in our study, further work is needed to increase seatbelt compliance to similar levels as those from developed countries such as Australia (97%), Canada (96%), and the UK (96%) (Chan 2013). Similar to goals set by international programs to increase seatbelt use, SA should aim to increase seatbelt compliance to 95% among drivers and passengers (Slyunkina et al. 2013).

The magnitude of improved compliance presented here may dissipate over time as in the case with enforcement strategies evaluated previously in developed countries (McCartt and Geary 2004; Rajalin et al. 2005). This is because we assessed the immediate effect a month after the implementation of the intervention. We opted to conduct the study shortly after the intervention went into effect as a major change in the driving environment is set to take place in less than 2 months, that is allowing women to drive motor vehicles for the first time (Hubbard 2017). As in the case of similar interventions, there is a need to conduct further studies to examine the sustainability of the effects identified in our study.

The new detection system is one of many programs supported by the *Saudi Vision 2030*, which is a national landmark plan aiming to improve population health and reduce disabilities (National Transformation Program 2016b). Among its many goals, the *Vision* aims to enact legislation, support police enforcement, and launch awareness campaigns to reduce RTIs. Our results may support the *Vision* by informing scientists, officials, and the public of the positive implications when investing in prevention. Moreover, our findings may pave the way to address other neglected areas of traffic safety such as risky driving behaviors. The fine for not wearing seatbelts and using mobile phones while driving is 150 SAR ($40). This may not represent enough penalty to discourage violators. Therefore, traffic police may evaluate whether increasing the fine is associated with significant reduction in traffic violations and associated crashes. A recent meta-analysis of the effect of increasing traffic violation fines revealed that increasing fine 50–100% is associated with increasing drivers’ compliance by 15%, and 5–10% reduction in the incidence of traffic crashes (Goldenbeld 2017). Further interventions to improve drivers’ compliance may include community opportunistic education, rewarding safe compliant drivers, and altering drivers’ desire of traffic violation (Hoekstra and Wegman 2011).

Despite lack of significance, our results showed some increase in mobile phone use following the intervention at some inner intersections. This is an interesting unexpected finding and it may be explained by several factors. First, this could be a reflection of the overall global trend of increased engagement with mobile phones and social media applications. Second, drivers may feel that enforcement is only limited to major highways, thus, they are unlikely to receive a violation. Finally, as seatbelt use becomes more prevalent, drivers may underestimate the risk of injury from distracted driving because of increased protection “Peltzman effect” (Lv et al. 2015). Further research is needed to explore the underlying causes and possible interventions to reduce mobile phone use among drivers.

This study has several strengths. It is the first study in SA to include multiple data points to assess and describe the impact of a new traffic intervention and by using control data collected prior to announcing its implementation. Our findings may facilitate further studies evaluating new interventions and measuring their effectiveness. In addition, we used a large sample of direct observations to assess compliance rather than self-reported measures, which have been found to overestimate the prevalence of seatbelt compliance (Özkan et al. 2012).

The study has several limitations that need to be considered when evaluating the present findings. First, the evaluation was performed approximately 1 month after the implementation, which may not represent a sufficient time to change drivers’ behavior. Nevertheless, as described above, the intervention was not implemented abruptly; thus, we believe drivers in Riyadh were fully aware of its implementation. Also, because the Saudi *Vision* includes many programs, it is possible that other initiatives may have contributed to the change in violation presented here. Second, due to funding limitations, we were not able to conduct a before-and-after analysis including a comparison group (i.e., another city) without an implemented system. Therefore, we cannot rule the potential impact of unmeasured confounders on improving compliance with seatbelt and mobile phone laws.

Third, data collection was conducted during a specified period during the daytime of working days, which may affect the generalizability of the findings. Fourth, we excluded observations from vehicles with a tinted windshield. Because this is a traffic violation by itself and it is possible we overestimated seatbelt use and underestimated mobile phone use while driving. However, the data collection approach was the same before and after cameras implementation reducing the likelihood of bias. Finally, due to the nature of the study, we were not able to evaluate the impact of certain variables on the likelihood of using a mobile phone or wearing a seatbelt such as age, education and previous violations.

## Conclusions

In summary, our study suggests that the newly implemented camera system is associated with significant increase in compliance with seatbelt and mobile phone use laws. The present findings may be used to engage the public to improve traffic safety and to advocate for further investment in public health interventions to reduce RTIs and improve population health. Seatbelt and mobile phone use while driving should continue to be monitored, and further enforcement efforts are needed in order to improve and maintain reduced levels of traffic violation and increase traffic safety.

## Abbreviations

IRB: Institutional Review Board
OR: Odds ratio
RTI: Road traffic injury
SA: Saudi Arabia
THI: Traumatic head injury
UK: United Kingdom
US: United States
YPLL: Years of potential life lost
